# Anaplastic thyroid carcinoma with bilateral internal jugular vein tumour thrombus formation: a case report

**DOI:** 10.1093/bjrcr/uaaf021

**Published:** 2025-03-22

**Authors:** Ruiqian Yan, Junxi Gao

**Affiliations:** Department of Ultrasound Diagnosis Abdomen, The First Affiliated Hospital of Xinjiang Medical University, Urumqi, Xinjiang 830011, China; Department of Ultrasound Diagnosis Abdomen, The First Affiliated Hospital of Xinjiang Medical University, Urumqi, Xinjiang 830011, China

**Keywords:** interstitial thyroid carcinoma, conventional ultrasound, jugular vein cancer thrombus

## Abstract

Anaplastic thyroid carcinoma (ATC) is a highly aggressive thyroid malignancy, comprising 1%-4% of thyroid cancers, with rapid local invasion and distant metastasis. We report a 57-year-old male with ATC presenting with a neck mass, hoarseness, and dysphagia. Imaging showed cancer thrombus in bilateral internal jugular veins, with a biopsy confirming ATC. This case highlights the imaging and pathological features of ATC and emphasizes the importance of ultrasound in evaluating intravascular cancer thrombus, aiding accurate diagnosis and management.

## Clinical presentation

A 57-year-old male patient, who found an anterior cervical mass 1 year ago, was admitted to our hospital 1 month ago because of a markedly enlarged anterior cervical mass accompanied by hoarseness and difficulty in swallowing.

## Imaging findings

**Figure 1.**
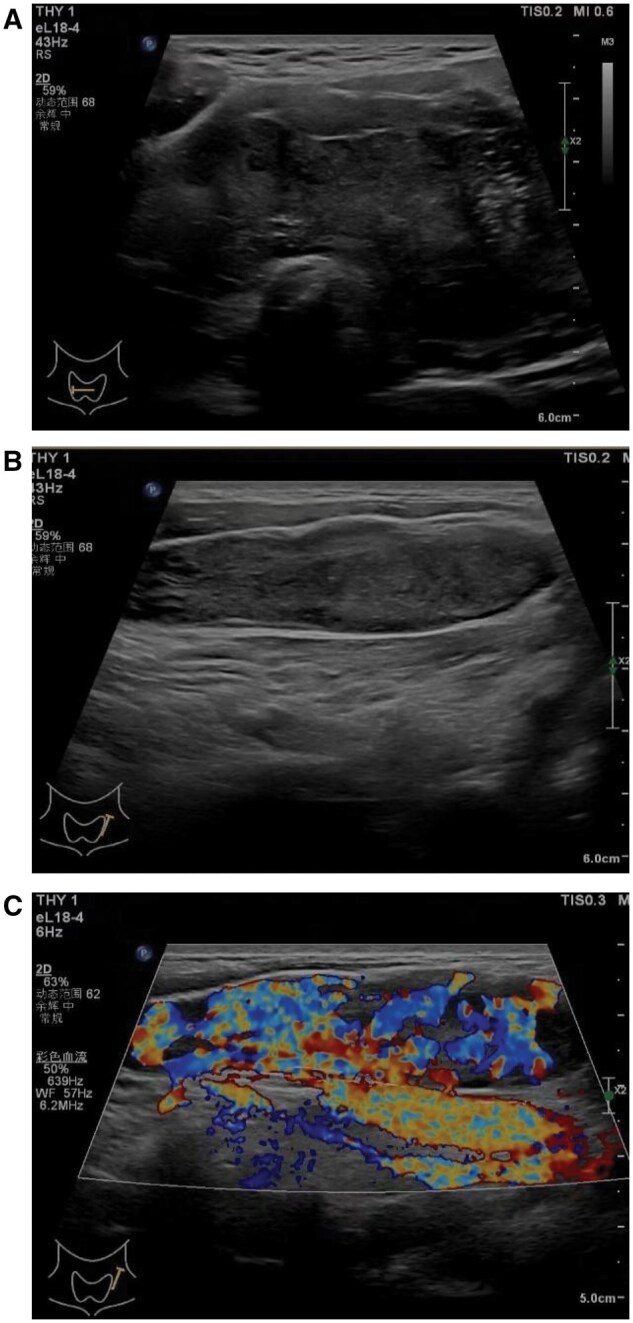
(A) Ultrasonography showed a markedly enlarged and diffusely diseased thyroid gland, with multiple microcalcifications and foci of circumferential calcification visible in the thyroid gland. (B) Hypoechoic filling is seen in the internal jugular vein, and the lumen cannot be deflated by probe compression. (C) Colour Doppler shows abundant blood flow signals in hypoechoic areas.

**Figure 2.**
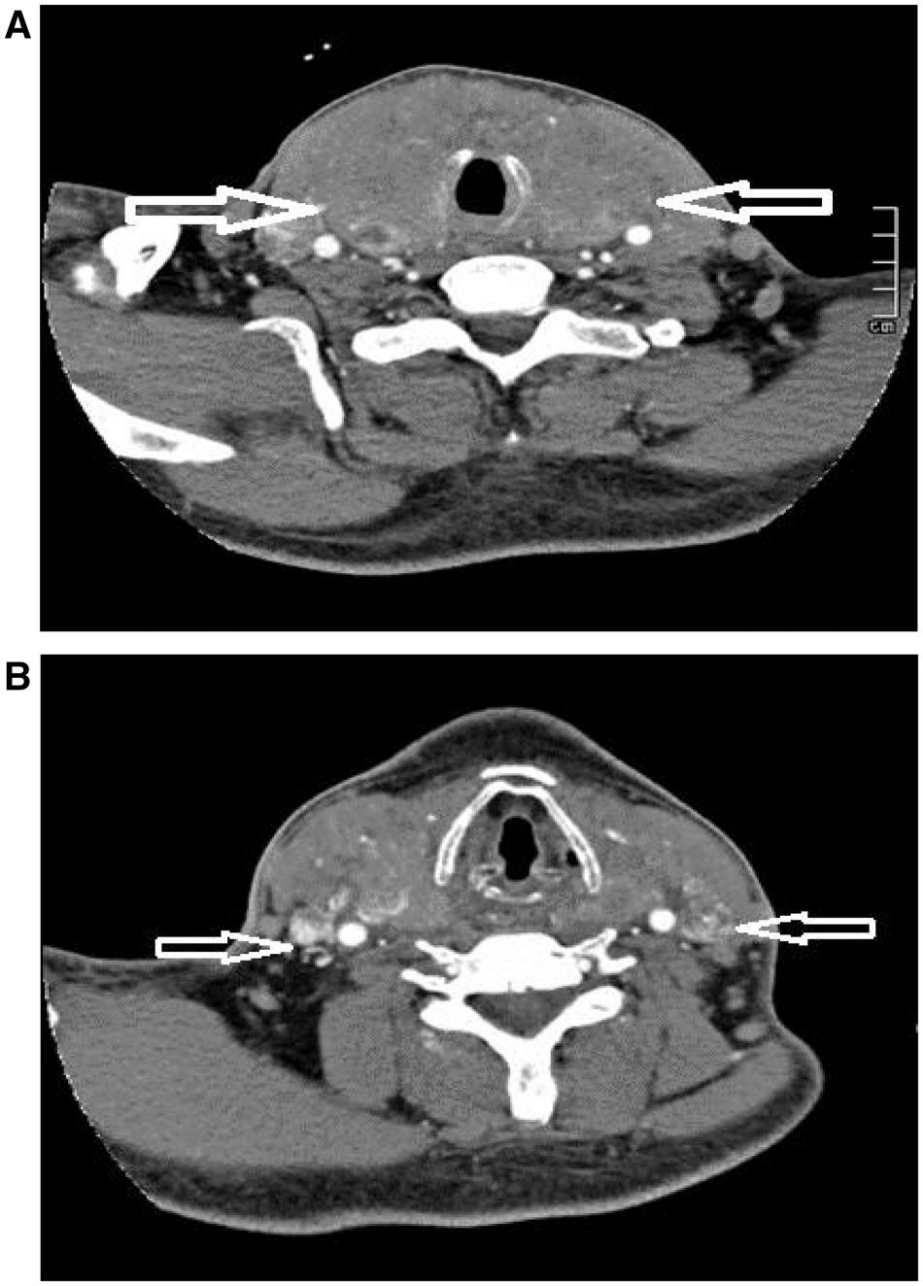
(A) Enhanced CT examination showed markedly enlarged thyroid volume and uneven enhancement of bilateral lobes, with circular and nodular calcified foci visible within. (B) Slightly hypodense shadows and nodular and irregularly shaped hypodense shadows are seen filling the lumen of the bilateral jugular vein tracts.

Ultrasound showed that the right lobe of the thyroid gland was about 4.2 cm thick, the left lobe was about 4.0 cm thick, and the isthmus was about 2.2 cm thick, with abnormal morphology; multiple strong echoes could be seen in the thyroid gland, some of which were in tiny clusters, some of them were in the shape of a ring, with no acoustic shadows at the back, and the rest of the thyroid gland tissues were hypoechoic; tortuous tubular hypoechoic echogenicity could be seen around the thyroid glands bilaterally, mainly in the isthmus, and the colour Doppler showed that hyperechogenicity could be seen in the form of point streaks of blood flow signals. The lumen of the internal jugular vein and its cervical branches was full of hypoechoic sound, and the lumen could not be completely deflated after probe pressure was applied, and the colour Doppler showed that the hypoechoic sound was richly distributed with blood flow signals (Figure 1). Ultrasound-guided coarse needle aspiration biopsy suggested a malignant tumour of the thyroid gland. Enhanced CT showed that the volume of the thyroid gland increased significantly and the enhancement of bilateral lobes was uneven, ring-shaped, and nodular calcification foci could be seen in the thyroid gland, the trachea was narrowed by compression, and the lumen of bilateral jugular veins was not uniformly densely packed, with slightly low-density shadows, and nodular and irregularly shaped high-density shadows could be seen (Figure 2).

## Treatment, outcome, and follow-up

**Figure 3.**
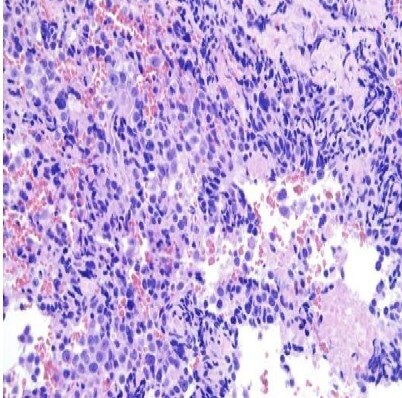
Postoperative pathology consistent with interstitial carcinoma of the thyroid gland (HE stain, ×20).

Radical thyroid cancer surgery was performed in the clinic. During the operation, the volume of the thyroid gland increased significantly, cystic and solid occupations were palpable in the thyroid gland, the tumour tissue invaded the surrounding muscles and blood vessels, and the blood vessels around the thyroid gland were thickened significantly. The tumour tissue pushed the internal jugular vein to cross and infiltrative growth to the vein. Under the microscope, the tumour tissue in the thyroid gland showed solid nest-like growth, with papillary structures in the local area, and the cell nuclei were easily seen (Figure 3). Immunohistochemistry: cytokeratin (CK) (+), thyroid transcription factor-1 (TTF-1) (+), paired box gene (Pax-8) (+), CD56 (+), VIM (+), Ki-67 (45%). Clinical diagnosis: interstitial thyroid carcinoma with necrosis and intravascular cancerous thrombosis.

## Discussion

Anaplastic thyroid carcinoma (ATC) is a highly aggressive malignant tumour of the thyroid, accounting for 1%-4% of all thyroid cancers and is more common in older adults. The disease progresses rapidly; literature reports indicate that approximately 40%-45% of patients have already metastasized to surrounding lymph nodes at the time of initial diagnosis.^1^^,^^2^ Direct invasion by ATC leading to bilateral internal jugular vein thrombosis is rarely reported. Most of these patients seek medical attention due to rapidly enlarging neck masses, presenting clinical symptoms such as hoarseness, difficulty swallowing, dyspnoea, and superior vena cava syndrome.^3^ Furthermore, once a thrombus forms, it not only obstructs venous return but may also lead to distant organ metastasis and even result in pulmonary embolism or right atrial obstruction, causing acute death in patients. In this case, the patient had cancer thrombi filling both the surrounding thyroid veins and the bilateral internal jugular veins, while the vessel walls of the bilateral internal jugular veins remained intact. The analysis suggests that thrombus formation occurs when tumour tissue first invades the surrounding venous drainage of the thyroid (such as the facial vein, superior thyroid vein, or middle thyroid vein) or the lumen of branches of the internal jugular vein, where it combines with fibrin and deposits within the vessel, allowing the thrombus to continue to extend and grow within the vasculature.

The diagnosis of ATC typically relies on a comprehensive assessment of imaging, cytology, and pathology. In this case, the ultrasound findings of the thyroid lesion showed significant enlargement and diffuse changes in the thyroid gland, with multiple tiny calcifications and ring-like calcified foci visible within the gland, consistent with reports by Panzironi et al.^4^ The diagnosis of ATC typically relies on a comprehensive assessment of imaging, cytology, and pathology. In this case, the ultrasound findings of the thyroid lesion showed significant enlargement and diffuse changes in the thyroid gland, with multiple tiny calcifications and ring-like calcified foci visible within the gland, consistent with reports by Panzironi et al. This condition must be differentiated from diffuse thyroid lymphoma and follicular thyroid carcinoma based on ultrasound characteristics. Patients with diffuse thyroid lymphoma often have associated Hashimoto’s thyroiditis, and a minority may present with symptoms such as fever and night sweats. The ultrasound appearance is usually characterized by unilateral or bilateral thyroid enlargement with diffuse changes, often asymmetrical between the lobes and may show linear high echogenicity. Most patients also exhibit abnormal lymph node structures in the bilateral neck and throughout the body.^5^ The ultrasound findings of follicular thyroid carcinoma typically present as solid isoechoic or hyperechoic nodules within the thyroid, with lesions having a complete capsule or clear boundaries, and the internal echogenicity is generally homogeneous. Lymph node involvement is rare, and distant metastases are commonly seen in the lungs and bones.^6^ The ultrasound appearance of cancer thrombus is characterized by low echogenicity filling the internal jugular veins and their branches, with colour Doppler imaging revealing abundant blood flow signals in the low echogenic areas, making it easy to differentiate from internal jugular vein thrombosis. Additionally, enhanced CT and MRI can effectively assess the local invasiveness of the tumour and potential metastases.^7^ The application of fine-needle aspiration biopsy combined with immunohistochemical techniques not only improves the diagnostic accuracy of thyroid lesions but also provides important references for personalized treatment.^8^ In summary, when performing ultrasound examinations on patients with thyroid tumours, ultrasound physicians should carefully examine the surrounding venous return and the internal jugular vein and its branches, paying attention to the presence of intravascular cancer thrombus.

## Learning points

- Ultrasonographers should be aware of the clinical and imaging manifestations of the disease to clarify the diagnosis as early as possible and to provide an imaging reference for the clinical staging of the tumour and the treatment strategy.
- When sonographers perform ultrasound testing on patients with thyroid tumours, they should carefully examine the perithyroid refluxing veins as well as the internal jugular vein and its collateral branches, noting the presence of intravascular cancerous emboli.

## Data Availability

Because this is a case report, there are no associated research data to be shared.
